# Clinical Findings of COVID-19 Patients Admitted to Intensive Care Units in Guangdong Province, China: A Multicenter, Retrospective, Observational Study

**DOI:** 10.3389/fmed.2020.576457

**Published:** 2020-10-19

**Authors:** Yonghao Xu, Zhiheng Xu, Xuesong Liu, Lihua Cai, Haichong Zheng, Yongbo Huang, Lixin Zhou, Linxi Huang, Yun Ling, Liehua Deng, Jianwei Li, Sibei Chen, Dongdong Liu, Zhimin Lin, Liang Zhou, Weiqun He, Nanshan Zhong, Xiaoqing Liu, Yimin Li

**Affiliations:** ^1^State Key Laboratory of Respiratory Diseases, Department of Critical Care Medicine, Guangzhou Institute of Respiratory Health, First Affiliated Hospital of Guangzhou Medical University, Guangzhou, China; ^2^Department of Critical Care Medicine, Dongguan People's Hospital, Dongguan, China; ^3^Department of Critical Care Medicine, Foshan First People's Hospital, Foshan, China; ^4^Department of Critical Care Medicine, The First Affiliated Hospital of Shantou University Medical College, Shantou, China; ^5^Department of Critical Care Medicine, Huizhou Municipal Central Hospital, Huizhou, China; ^6^Department of Critical Care Medicine, Affiliated Hospital of Guangdong Medical University, Zhanjiang, China; ^7^Department of Critical Care Medicine, Zhongshan City People's Hospital, Zhongshan, China

**Keywords:** intensive care unit, COVID-19, SARS-CoV-2, critically ill, mortality

## Abstract

**Background:** Information about critically ill patients with coronavirus disease 2019 (COVID-19) in China but outside of Wuhan is scarce. We aimed to describe the clinical features, treatment, and outcomes of patients with COVID-19 admitted to the intensive care unit (ICU) in Guangdong Province.

**Methods:** In this multicenter, retrospective, observational study, we enrolled consecutive patients with COVID-19 who were admitted to seven ICUs in Guangdong Province. Demographic data, symptoms, laboratory findings, comorbidities, treatment, and outcomes were collected. Data were compared between patients with and without intubation.

**Results:** A total of 45 COVID-19 patients required ICU admission in the study hospitals [mean age 56.7 ± 15.4 years, 29 males (64.4%)]. The most common symptoms at onset were fever and cough. Most patients presented with lymphopenia and elevated lactate dehydrogenase. Treatment with antiviral drugs was initiated in all patients. Thirty-six patients (80%) developed acute respiratory distress syndrome at ICU admission, and 15 (33.3%) septic shock. Twenty patients (44.4%) were intubated, and 10 (22.2%) received extracorporeal membrane oxygenation. The 60-day mortality was 4.4% (2 of 45).

**Conclusion:** COVID-19 patients admitted to ICU were characterized by fever, lymphopenia, acute respiratory failure, and multiple organ dysfunction. The mortality of ICU patients in Guangdong Province was relatively low with a small sample size.

## Introduction

In December 2019, human infection with a novel coronavirus, known as severe acute respiratory syndrome coronavirus disease 2 (SARS-CoV-2), was confirmed in Wuhan, China, and spread rapidly beyond Wuhan and around the world (1, 2). By May 1, 2020, a total of 84,388 patients were infected in mainland China, with 4,643 deaths, according to a Chinese Center for Disease Control and Prevention report (3). Previous studies have mainly focused on the general epidemiological findings, clinical presentation, and clinical outcomes of mild and moderately affected patients with coronavirus disease 2019 (COVID-19) (4–7). One recent study reported the characteristics of critically ill patients in a single center in Wuhan with 61.5% mortality (8). However, the clinical characteristics of patients with COVID-19 admitted to intensive care units (ICUs) in China but outside of Wuhan have not been described, including in Guangdong Province, where by May 1, 2020, more than 1,000 people had been confirmed as having COVID-19 (3). Here, we describe the characteristics and treatment of ICU patients with COVID-19 in Guangdong Province.

## Methods

### Study Design

This multicenter, retrospective, observational study was designed, and conducted by the First Affiliated Hospital of Guangzhou Medical University. Seven hospitals were involved: First Affiliated Hospital of Guangzhou Medical University, Dongguan People's Hospital, Foshan First People's Hospital, First Affiliated Hospital of Shantou University Medical College, Affiliated Hospital of Guangdong Medical University, Huizhou Municipal Central Hospital, and Zhongshan City People's Hospital. The details of the ICUs and infection control practices are provided in the Supplementary Appendix (Supplementary Table 1). The study was approved by the Ethics Commission of the First Affiliated Hospital of Guangzhou Medical University. The informed consent requirement was waived because the study was retrospective.

### Patients

Consecutive patients were enrolled from January 14, 2020, to February 20, 2020, and all had confirmed SARS-CoV-2 infection by real-time polymerase-chain-reaction testing of throat swab specimens. The illness severity of COVID-19 was defined according to the Chinese management guideline for COVID-19 (version 7.0) (9). Severe cases were defined as meeting any of the following criteria: (1) respiratory distress (≥30 breaths/ min); (2) oxygen saturation ≤ 93% at rest; (3) arterial partial pressure of oxygen (Pao_2_)/ fraction of inspired oxygen (Fio_2_) ≤ 300 mm Hg. Critical cases were defined as meeting any of the following criteria: (1) respiratory failure and requiring mechanical ventilation; (2) shock; (3) with other organ failure that required ICU care. In our study, the criteria for ICU admission included both the severe cases and critical cases. For the enrolled patients, their living status, intubation, weaning, and ICU and hospital discharge dates were confirmed on April 20, 2020.

### Data Collection

Medical records of patients who were in the ICU from January 14, 2020, to April 20, 2020, were extracted and sent to the data collection center in the First Affiliated Hospital of Guangzhou Medical University. A team of ICU doctors who had been treating patients with COVID-19 collected and reviewed the data. If information was not clear, the central working group contacted the doctor responsible for the treatment of the patient for clarification. Information recorded included demographic data, underlying conditions, symptoms, laboratory and chest radiograph findings, comorbidities, intubation rates, and ventilator settings prior to and during ICU therapy.

### Definition

Fever was defined as axillary temperature of at least 37.3°C. The incidence of COVID-19–related comorbidities was identified, including acute respiratory distress syndrome (ARDS), septic shock, cardiac injury, acute kidney injury, liver dysfunction, and gastrointestinal hemorrhage. ARDS was diagnosed according to the Berlin definition (10), and septic shock was identified by the Sepsis-3 definition (11). Acute kidney injury was identified on the basis of elevated serum creatinine according to the Kidney Disease: Improving Global Outcomes guideline (12). Cardiac injury was recognized by increased cardiac troponin I or electrocardiography abnormalities of non-specific ST-T waves (13, 14). Transaminitis was defined by aspartate aminotransferase or alanine aminotransferase levels >80 U/L. Gastrointestinal hemorrhage was identified by a positive fecal or gastric fluid occult blood test (15).

### Statistics

Continuous variables are presented as either mean ± standard deviation (SD) or median with interquartile range (IQR), in accordance with either normal or non-normal distributions. For categorical variables, the frequency and percentage of patients in each category were calculated. Differences between intubated and non-intubated patients were assessed with two-sample *t*-test or Wilcoxon rank-sum test, depending on parametric or non-parametric data for continuous variables and χ^2^ test for categorical variables. The Spearman correlation coefficient was used to test the correlations between clinical variables. All analyses were performed using SPSS 23.0 (SPSS Inc., Chicago, IL, USA). A *p* < 0.05 (two-sided) was considered statistically significant.

## Results

### Demographic, Epidemiologic, and Baseline Characteristics of the Patients

**Figure 1:**
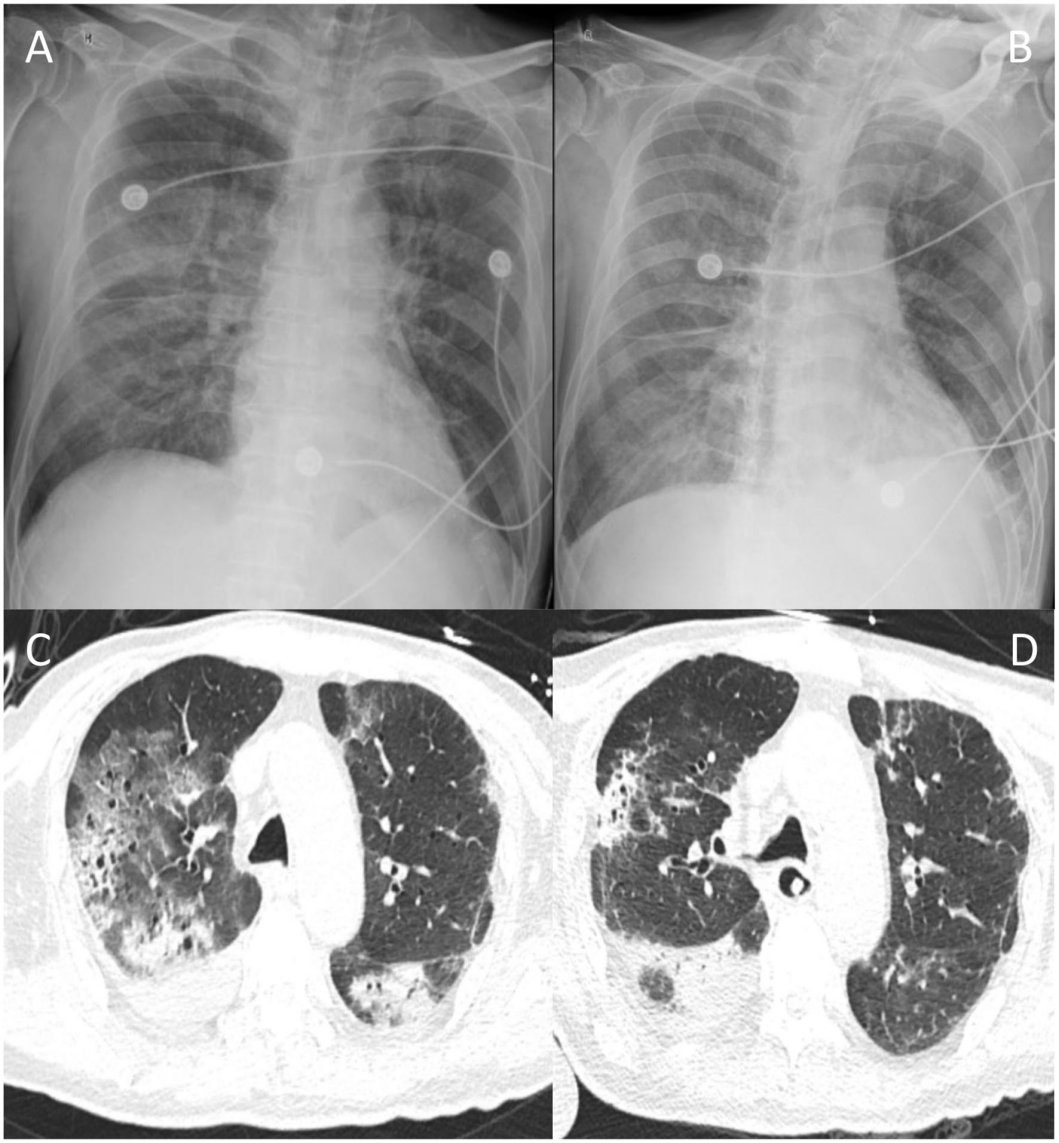
Typical chest radiographs of a patient with COVID-19. Chest radiographs of one patient on day 1 at ICU admission **(A)** and day 5 **(B)**; CT scan of chest of the same patient on day 10 **(C)** and day 20 **(D)** after ICU admission.

Of 332 patients with COVID-19, 45 patients requiring ICU admission were identified in the study hospitals. The first case included was confirmed on January 14, 2020, and the last one was confirmed on February 20, 2020. All patients had positive throat swabs for SARS-CoV-2 and bilateral infiltrates on chest radiographs (Figure 1) and were admitted to the ICU.

The mean age of the patients was 56.7 years (SD, 15.4), and 29 (64.4%) were men. A total of 26 patients (57.8%) had at least one preexisting condition, including hypertension (46.7%) and diabetes (28.9%). Thirty-six patients had a history of exposure to Hubei Province, and 26 (57.8%) had exposure to patients with COVID-19. Moreover, 19 patients (42.2%) had exposure to a familial cluster (Table 1).

**Table 1:** Demographics and baseline characteristics of patients with severe COVID-19.

**Characteristics**	**All patients (*n* = 45)**	**Intubated (*n* = 20)**	**Not intubated (*n* = 25)**
Age (years)	56.7 ± 15.4	62.1 ± 13.56	52.4 ± 15.7
**Sex**, ***n*** **(%)**			
Male	29 (64.4)	14 (70)	15 (60)
Female	16 (35.6)	6 (30)	10 (40)
Body mass index (kg/m^2^)	24.2 (22.0–26.7)	23.2 (21.4–25.3)	25.0 (22.9–26.9)
**Exposure**, ***n*** **(%)**			
Exposure to Hubei	36 (80.0)	18 (90)	18 (72)
Exposure to confirmed patients	26 (57.8)	12 (60)	14 (56)
Familial cluster	19 (42.2)	11 (55)	8 (32)
Positive reverse transcriptase–polymerase chain reaction, *n* (%)	45 (100)	20 (100)	25 (100)
**Chronic diseases**, ***n*** **(%)**			
At least one preexisting condition	26 (57.8)	14 (70)	12 (48)
Hypertension	21 (46.7)	11 (55)	10 (40)
Diabetes	13 (28.9)	6 (30)	7 (28)
Chronic cardiac disease	6 (13.3)	4 (20)	2 (8)
Chronic pulmonary disease	4 (8.9)	3 (15)	1 (4)
Cancers	3 (6.7)	1 (5)	2 (8)
Immunosuppression	1 (2.2)	0	1 (4)
Smoker (+ex-smoker), *n* (%)	8 (17.8)	3 (15)	5 (20)
**Onset symptoms (%)**			
Fever	39 (86.7)	18 (90)	21 (84)
Cough	32 (71.1)	15 (75)	17 (68)
Sputum	18 (40.0)	7 (35)	11 (44)
Dyspnea	29 (64.4)	15 (75)	14 (56)
Chest tightness	10 (22.2)	6 (30)	4 (20)
Myalgia	4 (8.9)	2 (10)	2 (8)
Malaise	17 (37.8)	10 (50)	7 (28)
Diarrhea	0	0	0
Duration from onset of symptoms to ICU admission (days)	10.0 (8.0–13.0)	11.0 (7.5–14.0)	10.0 (8.0–12.0)

### Clinical Characteristics and Laboratory Findings

The most common symptoms were fever (86.7%), cough (71.1%), and dyspnea (64.4%). The median time from onset of symptoms to ICU admission was 10 days (IQR, 8–13 days) (Table 1). The median Acute Physiology and Chronic Health Evaluation II (APACHE II) and Sequential Organ Failure Assessment (SOFA) scores of all patients at ICU admission were 14 (8–18) and 4.0 (3.0–6.8), respectively. A total of 20 patients (44.4%) were intubated within 3 days of their ICU admission.

White blood cell counts were in the normal range with neutrophils predominant in intubated patients. Forty-one patients (91.1%) presented with lymphopenia (lymphocytes <1.0 × 10^9^/L). Compared with non-intubated patients, those intubated showed a significant decrease in lymphocyte counts [0.7 (0.5–0.9) vs. 0.4 (0.3–0.5), *p* < 0.001)]. Elevated levels of lactate dehydrogenase (LDH) (normal range, 109–255 U/L) were observed in 32 patients (71.1%). Additionally, intubated patients had higher levels of LDH than non-intubated patients [397.1 (342.2–523.8) vs. 285.3 (215.5–346.7), *p* = 0.0012]. Spearman correlation analyses showed that SOFA scores were negatively correlated with lymphocyte count and positively associated with LDH (Spearman ρ = −0.57 and 0.51, respectively, *p* < 0.001) (Supplementary Figure 1). Prothrombin time, d-dimer, troponin I, creatine kinase, serum creatinine, aspartate aminotransferase, lactate, procalcitonin, and potassium were significantly increased in intubated patients, whereas Pao_2_/Fio_2_ ratio, hemoglobin, and platelet count were lower in intubated than non-intubated patients (Table 2).

**Table 2:** Differences in Laboratory findings in patients with severe COVID-19.

**Variables**	**All patients (*n* = 45)**	**Intubated (*n* = 20)**	**Not intubated (*n* = 25)**	***P***
Pao_2_/Fio_2_ ratio (mm Hg)	170.0 (115.5–218.2)	130.6 (100.2–197.0)	198.8 (142.4–279.1)	0.0030
Paco_2_ (mm Hg)	38.3 (35.5–41.5)	40.3 (34.5–47.6)	38.0 (35.1–39.7)	0.1142
White blood cell count ( ×10^9^/L)	8.3 (5.6–10.3)	9.7 (7.4–12.9)	6.5 (4.8–9.2)	0.0037
Neutrophil count ( ×10^9^/L)	6.8 (4.4–9.5)	9.0 (6.330–13.15)	5.5 (3.8–8.3)	0.0022
Lymphocyte count ( ×10^9^/L)	0.5 (0.3–0.7)	0.4 (0.3–0.5)	0.7 (0.5–0.9)	<0.001
Hemoglobin (g/L)	121.0 (111.0–133.0)	115.5 (105.5–122.5)	129.0 (116.5–141.0)	0.0024
Platelet count ( ×10^9^/L)	189.7 ± 72.7	165.5 ± 51.5	209.0 ± 81.9	0.0448
Prothrombin time (s)	13.4 (12.3–14.9)	14.6 (13.2–15.2)	13.1 (12.0–13.7)	0.0341
Activated partial thromboplastin time (s)	35.8 (28.4, 41.4) (*n* = 37)	38.9 (35.1, 46.9) (*n* = 18)	32.1 (26.0,36.0) (*n* = 19)	0.0880
Fibrinogen (g/L)	4.61 (3.61, 5.62) (*n* = 37)	4.92 (3.67, 6.12) (*n* = 18)	4.59 (3.8, 5.1) (*n* = 19)	0.1510
d-Dimer (ng/mL)	980 (556, 1,807) (*n* = 35)	1,490 (859, 2,297) (*n* = 18)	741 (4,480, 1,231) (*n* = 17)	0.0235
Troponin I (μg/L)	0.01 (0.01–0.02) (*n* = 40)	0.03 (0.02–0.05) (*n* = 20)	0.01 (0.00–0.01) (*n* = 20)	<0.001
Lactate dehydrogenase (U/L)	338.0 (248.0–437.9)	397.1 (342.2–523.8)	285.3 (215.5–346.7)	0.0012
Creatine kinase (U/L)	66.5 (44.7–114.4) (*n* = 43)	83.0 (52.2–221.2) (*n* = 20)	45.0 (30.6–75.4) (*n* = 23)	0.0034
Serum creatinine (μmol/L)	67.6 (54.2–86.0) (*n* = 44)	75.6 (61.8–116.0) (*n* = 20)	62.7 (53.3–78.9) (*n* = 24)	0.0305
Total bilirubin (μmol/L)	15.5 (10.5–21.3)	12.5 (9.1–20.6)	19.0 (13.2–22.7)	0.1311
Aspartate aminotransferase (U/L)	27 (22.0–39.5) (*n* = 44)	32.4 (24.8–60.1) (*n* = 20)	23.1 (20.3–32.8) (*n* = 24)	0.0261
Alanine aminotransferase (U/L)	29.0 (20.1–50.0)	27.3 (17.6–46.8)	30.0 (19.7–56.0)	0.5831
Albumin (g/L)	31.6 (30.2–34.5)	31.0 (30.0–34.5)	33.0 (30.3–35.4)	0.7472
Lactate (mmol/L)	1.9 (1.5–2.3) (*n* = 40)	2.1 (1.7–2.7) (*n* = 20)	1.5 (1.3–2.1) (*n* = 20)	0.0145
Procalcitonin (ng/mL)	0.1 (0.1–0.3)	0.4 (0.1–2.9)	0.1 (0.1–0.1)	<0.001
Potassium (mmol/L)	3.9 ± 0.7	4.2 ± 0.8	3.7 ± 0.5	0.0196
Sodium (mmol/L)	137.2 ± 4.0	138.2 ± 5.0	136.3 ± 2.8	0.1081

*All the values were taken at the ICU admission*.

According to Berlin definition, 36 patients (80%) were diagnosed with ARDS at ICU admission, 5 (13.9%) with mild ARDS, 22 (61.1%) with moderate ARDS, and 9 (25%) with severe ARDS. Most patients had developed organ function damage during ICU stay, including 15 (33.3%) with septic shock, 8 (17.8%) with acute kidney injury, 15 (33.3%) with cardiac injury, 28 (62.2%) with transaminitis, 14 (31.1%) with gastrointestinal hemorrhage, and 4 (8.9%) with barotrauma. Secondary infections, including bacterial coinfection and fungal coinfection, were identified in 19 (42.2%) and 18 (40%) patients, respectively (Table 3).

**Table 3:** Comorbidities in ICU and treatments of patients with severe COVID-19.

**Characteristics**	**All patients (*n* = 45)**	**Intubated (*n* = 20)**	**Not intubated (*n* = 25)**
APACHE II score	14.0 (8.0–18.0)	18.0 (15.0–24.8)	10.0 (7.0–13.0)
SOFA score	4.0 (3.0–6.8)	6.0 (5.0–11.0)	3.0 (2.0–3.8)
**Comorbidities in ICU**, ***n*** **(%)**			
ARDS	36 (80)	20 (100)	16 (64)
Mild, P/F ratio (mm Hg)	250 (218–256) (*n* = 5)	218 (*n* = 1)	253 (238–257) (*n* = 4)
Moderate	126 (105–163) (*n* = 22)	115 (105–127) (*n* = 10)	149 (112–180) (*n* = 12)
Severe	90 (81–94) (*n* = 9)	90 (81–94) (*n* = 9)	0
Septic shock	15 (33.3)	15 (75)	0
Cardiac injury	15 (33.3)	13 (65)	2 (8)
Acute kidney injury	8 (17.8)	8 (40)	0
Transaminitis	28 (62.2)	16 (80)	12 (48)
Gastrointestinal hemorrhage	14 (31.1)	12 (60)	2 (8)
Barotrauma	4 (8.9)	4 (20)	0
Bacterial coinfection	19 (42.2)	15 (75)	4 (16)
Fungal coinfection	18 (40)	11 (55)	7 (28)
**Treatment in ICU**, ***n*** **(%)**			
Antiviral agents	45 (100)	20 (100)	25 (100)
Antibacterial agents	45 (100)	20 (100)	25 (100)
Antifungal agents	19 (42.2)	14 (70)	5 (20)
Convalescent plasma	6 (13.3)	6 (30)	0
Glucocorticoids	28 (62.2)	13 (65)	15 (60)
Immunoglobulin	28 (62.2)	15 (75)	13 (52)
Albumin	35 (77.8)	18 (90)	17 (68)

*P/F ratio was available at ICU admission*.

### Treatment in ICU

All patients received antiviral and antibacterial therapy. A total of 14 patients received oseltamivir, 29 with ribavirin, 22 with α-interferon, 24 with lopinavir–ritonavir, and 21 with arbidol (Supplementary Table 2). Antifungal agents were given to 19 patients (42.2%). A total of 28 patients (62.2%) had received glucocorticoids, 28 (62.2%) had received immunoglobulin, and 35 (77.8%) had received albumin (Table 3). Convalescent plasma was applied in six critically ill patients (13.3%), and no transfusion reactions occurred (Supplementary Table 2).

Of the 37 patients (82.2%) treated with a high-flow nasal cannula, 6 failed and received non-invasive mechanical ventilation; 13 failed and were intubated. Of the 17 patients (37.8%) with non-invasive mechanical ventilation, 7 failed and were intubated. Thus, 20 patients (44.4%) received invasive mechanical ventilation. For intubated patients, tidal volumes of 7.0 mL/kg predicted body weight were applied in accordance with lung protective ventilation strategy (16). Recruitment maneuvers were administered in six patients (30%). Five patients (25%) received prone position ventilation and extracorporeal membrane oxygenation (ECMO) and continuous renal replacement therapy (CRRT) were applied in 10 (22.2%) and 5 (11.1%) patients, respectively. Fifteen patients (33.3%) were administered vasoconstrictive agents, 20 (44.4%) with sedation, and analgesia and 8 (17.8%) received neuromuscular-blocking agents (Table 4).

**Table 4:** Respiratory settings of patients with severe COVID-19.

**Respiratory settings**	**All patients (*n* = 45)**
Oxygen therapy by mask/nasal cannula (%)	6 (13.3)
Mask/nasal cannula to HFNC (%)	6 (100)
Mask/nasal cannula to NIV (%)	0
HFNC (%)	37 (82.2)
Flow rates (L/min)	50 (40–50)
Fio_2_ (%)	50 (45–60)
HFNC to NIV (%)	6 (16.2)
HFNC to intubation (%)	13 (35.1)
Duration of HFNC before intubation (day)	2.5 (1.0–5.3)
Non-invasive mechanical ventilation (%)	17 (37.8)
BiPAP (%)	17 (100)
Fio_2_ (%)	42.5 (40–50)
NIV to intubation (%)	7 (41.2)
Duration of NIV before intubation (day)	1 (1, 3)
Invasive mechanical ventilation (%)	20 (44.4)
A/C (%)	17 (85)
SIMV (%)	3 (15)
Tidal volume, PBW (mL/kg)	7.00 ± 0.59 (*n* = 14)
PEEP (cm H_2_O)	10.0 (9.0–10.5)
Fio_2_ (%)	67.5 (60.0–71.3)
Lung recruitment (%)	6 (30)
Prone position ventilation (%)	5 (25)
Sedation and analgesia (%)	20 (44.4)
Neuromuscular blocking agents (%)	8 (17.8)
Vasoconstrictive agents (%)	15 (33.3)
ECMO (%)	10 (22.2)
CRRT (%)	5 (11.1)
Weaning from intubation before April 20 (%)	14 (70)
Weaning from ECMO before April 20 (%)	6 (60)
ICU discharge before April 20 (%)	35 (77.8)
ICU stay	17.5 (10.3–32.8) (*n* = 35)
Hospital discharge before April 20 (%)	31 (68.9)
Hospital stay	24.0 (20.0, 37.5) (*n* = 31)
60-day mortality (%)	2 (4.4)

*All values were taken at intubation or at the time receiving NIV or HFNC*.

*A/C, assist-control ventilation; SIMV, synchronized intermittent mandatory ventilation; PEEP, positive end-expiratory pressure; CPAP, continuous positive airway pressure; BiPAP, bilevel positive airway pressure; ECMO, extracorporeal membrane oxygenation; CRRT, continuous renal replacement therapy; ICU, intensive care unit. NIV, Non-invasive ventilation; HFNC, High-flow Nasal Cannula*.

### Clinical Outcomes

As of April 20, 2020, 14 patients (70%) were successfully weaned from invasive ventilation. Of 10 patients on ECMO, 6 were weaned, 2 patients died, and 2 were remained on ECMO. A total of 35 patients (77.8%) had been discharged from ICU, and 31 patients (68.9%) had recovered and were discharged from hospital. The 60-day mortality was 4.4% (2 of 45) (Table 4).

To identify the potential risks for intubation in patients with COVID-19, a χ^2^ test showed that older age (age ≥60 years), higher SOFA (≥4) and APACHE II (≥15) scores, high LDH (≥255 U/L), and lower lymphocyte count ( ≤ 0.8 × 10^9^/L) at ICU admission were associated with a higher risk for intubation (Supplementary Table 3).

## Discussion

This study presents a multicenter cohort of 45 patients admitted to ICUs for COVID-19 outside of Wuhan. Of all included patients, 36 (80%) developed ARDS at ICU admission, 20 (44.4%) required invasive mechanical ventilation, and 10 (22.2%) required ECMO, demonstrating that SARS-CoV-2 can cause severe illness.

Our study population had many of the clinical characteristics of patients with COVID-19. Lymphocytopenia occurred in more than 90% of COVID-19 patients admitted to ICU, which was similar to the Wuhan cohort (8). We found that intubated patients had significantly lower lymphocyte counts than non-intubated patients. We found that patients with lower lymphocyte counts were at higher risk for intubation and that lymphocyte counts were significantly negatively correlated with SOFA score at ICU admission. Lymphocytopenia is common in viral pneumonia, particularly in SARS and Middle East respiratory syndrome (MERS) (17, 18). It was reported that a lymphocyte count <0.8 × 10^9^/L was an independent risk factor for 90-day mortality in viral pneumonia (19). Therefore, lymphocytopenia may reflect the severity of COVID-19. Dynamic monitoring of the lymphocyte counts might be useful in terms of prognosis during the intensive care phase of the critical illness.

Although our patients were treated with antiviral drugs, including oseltamivir and lopinavir–ritonavir, to date no effective antiviral to treat COVID-19 has been identified. The current approach to clinical management is general supportive, supplemented with critical care and organ support when necessary (20, 21). It has been suggested that the administration of high-titer anti-influenza immune plasma derived from convalescent or immunized individuals may be clinically beneficial for the treatment of SARS, MERS, and seasonal influenza (22–24). Convalescent plasma is a potential treatment in coronavirus infection and has been suggested for use in COVID-19 (25). Therefore, convalescent plasma of patients with COVID-19 was administered to six patients (13.3%) in our cohort, and no transfusion reactions occurred. However, our findings cannot provide evidence of the efficacy of convalescent plasma in critically ill patients with COVID-19 because of limited sample sizes, short observation times, and lack of a randomized controlled group. Thus, treatment for the current outbreak of COVID-19 via convalescent plasma, particularly in patients with critical illness, should be carefully considered before well-designed clinical trials are conducted.

The 60-day mortality of ICU patients with COVID-19 was 4.4% (2 of 45) in our cohort, which was lower as compared to reported mortality of ICU patients in Wuhan and around the world (8, 26–29). There are a couple of factors associated with lower mortality. First, the numbers of infected cases and mortality rates related to COVID-19 vary from country to country. There is evidence that SARS-CoV-2 has undergone genetic changes in the process of spreading to other parts of the world after affecting China (30). A SARS-CoV-2 mutation was identified that was thought to have created a more “aggressive” form of the virus (31). Moreover, SARS-CoV-2 genomic variations were also reported to be associated with a higher mortality rate of COVID-19 (32–34). Therefore, fewer genetic changes of SARS-CoV-2 in the early beginning of China may account for the relative low mortality in China and in our study. Second, an intensivists-led multidisciplinary team was established and participated in combatting the COVID-19 pandemics at the very beginning of outbreak in the seven hospitals. A well-designed Web-based video consultation system for critically ill patients with COVID-19 was established and applied across different cities in Guangdong Province. This allowed a multidisciplinary team shared their experience and help with the management of critical illness remotely across hospitals. Third, a rapid response for patients with sudden clinical deterioration was also established to identify patients who developed into severe case and admit them to ICU for further monitor and interventions in a timely manner. The relatively lower APACHE II and SOFA scores (the median APACHE II and SOFA scores were 14 and 4.0, respectively) of all patients at ICU admission indicating a proportion of less critically ill patients were admitted to ICU to receive high-intensity monitor and prompt intensive care interventions. Fourth, the lack of ICU beds and invasive mechanical ventilators, patients management by a non-intensivists-dominated medical team may contribute to a high mortality in the epicenter of the coronavirus outbreak (35). Therefore, the high mortality of the patients (8, 26–29) may reflect the crisis of critical care medicine rather than the nature of COVID-19. With great difference, the relatively smaller number of patients in Guangdong Province, adequate ICU beds, and nurse-to-patient ratio (Supplementary Appendix) may partly contributed to a low mortality. Treatments, including convalescent plasma, invasive mechanical ventilation, CRRT, and ECMO, were applied for critically ill patients. Taken together, the mortality was relatively lower in ICU patients with COVID-19 in our cohort.

### Limitations

Our study has several limitations. First, the study included only 45 COVID-19 patients admitted to ICU from Guangdong Province. The conclusion about the small mortality may be inadequate because of a small sample. More clinical features related to the critical illness may have been identified if a larger sample size had been studied. Second, at the time we wrote this article, a small number of patients had not yet been discharged from ICU, so we were unable to document the exact length of ICU stay, the number of ventilation-free days, the case fatality rate, or the predictors of fatality. A longer follow-up period is needed. Third, the low number of patients prevents performing further statistical analysis such as multivariable logistic regression for independent predictors of intubation.

## Conclusions

The patients with COVID-19 from Guangdong Province in our study had a lower ICU mortality (2 of 45, 4.4%) but with a small sample size (*n* = 45). Larger cohort was still needed in future studies. Current treatment for critically ill patients with COVID-19 consists of appropriate support in ICU and careful monitoring until effective drugs are developed.

## Data Availability Statement

All datasets generated for this study are included in the article/Supplementary Material.

## Ethics Statement

The studies involving human participants were reviewed and approved by the Ethics Commission of the First Affiliated Hospital of Guangzhou Medical University. Written informed consent for participation was not required for this study in accordance with the national legislation and the institutional requirements.

## Author Contributions

XiL, YX, NZ, and YLi: conception and design. YLi, XiL, and NZ: administrative support. LC, HZ, YH, LixZ, LH, YLin, LD, JL, SC, DL, ZL, LiaZ, and WH: provision of study materials or patients. ZX, XuL, HZ, and YH: collection and assembly of data. YX, ZX, XuL, and LC: manuscript writing. All authors: data analysis, interpretation, and final approval of manuscript.

## Conflict of Interest

The authors declare that the research was conducted in the absence of any commercial or financial relationships that could be construed as a potential conflict of interest.
