# Supplementary material for: Clinical Findings of COVID-19 Patients Admitted to Intensive Care Units in Guangdong Province, China: A Multicenter, Retrospective, Observational Study
Source: Front Med (Lausanne). 2020 Oct 19;7:576457. doi: 10.3389/fmed.2020.576457 (PMC7604321; doi:10.3389/fmed.2020.576457)
Supplement: Supplementary file 1 [file Data_Sheet_1.docx]

**Supplementary Appendix**

**Clinical findings of COVID-19 patients admitted to intensive care units in Guangdong Province, China: a multi-center, retrospective, observational study**

Yonghao Xu^#1^, Zhiheng Xu^#1^, Xuesong Liu^#1^, Lihua Cai^#2^, Haichong Zheng^1^, Yongbo Huang^1^, Lixin Zhou^3^, Linxi Huang^4^, Yun Ling^5^, Liehua Deng^6^, Jianwei Li^7^, Sibei Chen^1^, Dongdong Liu^1^, Zhimin Lin^1^, Liang Zhou^1^, Weiqun He^1^, Nanshan Zhong^1^, Xiaoqing Liu*^1^, Yimin Li*^1^

^#^These authors contributed equally to this work.

**Institution**

1 State Key Laboratory of Respiratory Diseases, Guangzhou Institute of Respiratory Health, First Affiliated Hospital of Guangzhou Medical University, Department of Critical Care Medicine

2 Dongguan People's Hospital, Department of Critical Care Medicine

3 Foshan First People's Hospital, Department of Critical Care Medicine

4 The First Affiliated Hospital of Shantou University Medical College, Department of Critical Care Medicine

5 Huizhou Municipal Central Hospital, Department of Critical Care Medicine

6 Affiliated Hospital of Guangdong Medical University, Department of Critical Care Medicine

7 Zhongshan City People's Hospital, Department of Critical Care Medicine

**Supplementary Appendix**

At the 1 February 2020, a COVID-19 Study Group including seven hospitals which were designated hospitals for severe patients, was established. The seven hospitals involved were mainly for severe patients and managed by a multidisciplinary team lead by intensivists.

The department of intensive care unit (ICU) of the First Affiliated Hospital of Guangzhou Medical University had a closed unit with 16 beds, 4 single rooms with negative pressure and a nurse-to-patient ratio of 5.5:1. The ICU of Dongguan People's Hospital had a closed unit with 30 beds, 8 single rooms, 4 negative pressure rooms and a nurse-to-patient ratio of 2.4:1. The ICU of Foshan First People's Hospital had a closed unit with 12 beds, 6 single rooms with negative pressure and a nurse-to-patient ratio of 2.7:1. The ICU of First Affiliated Hospital of Shantou University Medical College had a closed unit with 20 beds, 4 single rooms and 2 negative pressure rooms and a nurse-to-patient ratio of 3:1. The ICU of Huizhou Municipal Central Hospital had a closed unit with 6 beds, 2 single rooms with negative pressure and a nurse-to-patient ratio of 3:1. The ICU of Affiliated Hospital of Guangdong Medical University had a closed unit with 8 beds, 4 single rooms, 2 negative pressure rooms and a nurse-to-patient ratio of 3:1. The ICU of Zhongshan City People's Hospital had a closed unit with 6 beds and all are single rooms with negative pressure with a nurse-to-patient ratio of 6:1.

**Infection control practices**

Strict isolation and protection measures were a top priority. Generally, confirmed patients were first isolated in single rooms with negative pressure. If more patients come, they were placed and treated in a closed unit. ICU staff were well trained with handwashing, environmental cleaning, and had adequate personal protective equipment. A standard practice was established during intubation. An expert who was expert in the procedure was recommended for intubation. Intubation was done in an airborne infection isolation room. Personal protective equipment, including protective clothing, head coverings, double-gloving, N95 respirator and eye protection, were provided during intubation.

Table S1 Distribution of patients per hospital

| Area of origin | All patients  (n=45) | Intubated  (n=20) | Not intubated  (n=25) |
| --- | --- | --- | --- |
| First Affiliated Hospital of Guangzhou Medical University | 16 (35.6) | 12 (60) | 4 (16) |
| Dongguan People’s Hospital | 10 (22.2) | 4 (20) | 6 (24) |
| Foshan First People’s Hospital | 9 (20.0) | 2 (10) | 7 (28) |
| First Affiliated Hospital of Shantou University Medical College | 3 (6.7) | 1 (5) | 2 (8) |
| Affiliated Hospital of Guangdong Medical University | 3 (6.7) | 0 | 3 (12) |
| Huizhou Municipal Central Hospital | 3 (6.7) | 0 | 3 (12) |
| Zhongshan City People’s Hospital | 1 (2.1) | 1 (5) | 0 |

Table S2 Treatment of antiviral and convalescent plasma

| Treatment | All Patients (n=45) |
| --- | --- |
| Antiviral, n (%) | 45 |
| Oseltamivir | 14 (31.1) |
| Ribavirin/ umifenovir | 29 (64.4) |
| ɑ-interferon | 22 (48.9) |
| Lopinavir-Ritonavir | 24 (53.3) |
| Arbidol | 21 (46.7) |
| Convalescent plasma, n (%) | 6 (13.3) |
| Serious adverse reaction |  |
| Allergic reaction | 0 |
| Acute haemolytic transfusion reaction | 0 |
| Febrile nonhaemolytic transfusion reaction | 0 |
| Transfusion related acute lung injury | 0 |
| Transfusion associated circulatory overload | 0 |
