# Supplementary material for: Clinical Findings of COVID-19 Patients Admitted to Intensive Care Units in Guangdong Province, China: A Multicenter, Retrospective, Observational Study
Source: Front Med (Lausanne). 2020 Oct 19;7:576457. doi: 10.3389/fmed.2020.576457 (PMC7604321; doi:10.3389/fmed.2020.576457)
Supplement: Supplementary file 2 [file Table_3.docx]

Table S3 Risk Factors for intubation

| Risk Factors | Intubated (n=20) | Not intubated (n=25) | P value |
| --- | --- | --- | --- |
| Age, years |  |  | 0.0275 |
| >60 | 13 | 8 |  |
| <60 | 7 | 17 |  |
| Preexisting conditions |  |  | 0.2244 |
| Yes | 14 | 12 |  |
| No | 6 | 13 |  |
| SOFA score at ICU admission |  |  | <0.0001 |
| > 4 | 18 | 7 |  |
| < 4 | 2 | 18 |  |
| APACHE II score at ICU admission |  |  | <0.0001 |
| > 15 | 18 | 3 |  |
| < 15 | 2 | 22 |  |
| PaO_2_/FiO_2_ at ICU admission |  |  | 0.0662 |
| >150 | 9 | 18 |  |
| <150 | 11 | 7 |  |
| Lymphocyte, (*10^9^/L) |  |  | 0.0165 |
| <0.8 | 20 | 17 |  |
| >0.8 | 0 | 8 |  |
| LDH, U/L |  |  | 0.0368 |
| > 255 | 17 | 14 |  |
| < 255 | 3 | 11 |  |

APACHE II score, Acute Physiology and Chronic Health Evaluation II score; SOFA, Sequential Organ Failure Assessment; PaO_2_/FiO_2_; LDH, lactate dehydrogenase
