# Supplementary figures and images for: Clinical Findings of COVID-19 Patients Admitted to Intensive Care Units in Guangdong Province, China: A Multicenter, Retrospective, Observational Study
Source: Front Med (Lausanne). 2020 Oct 19;7:576457. doi: 10.3389/fmed.2020.576457 (PMC7604321; doi:10.3389/fmed.2020.576457)

Figure S1

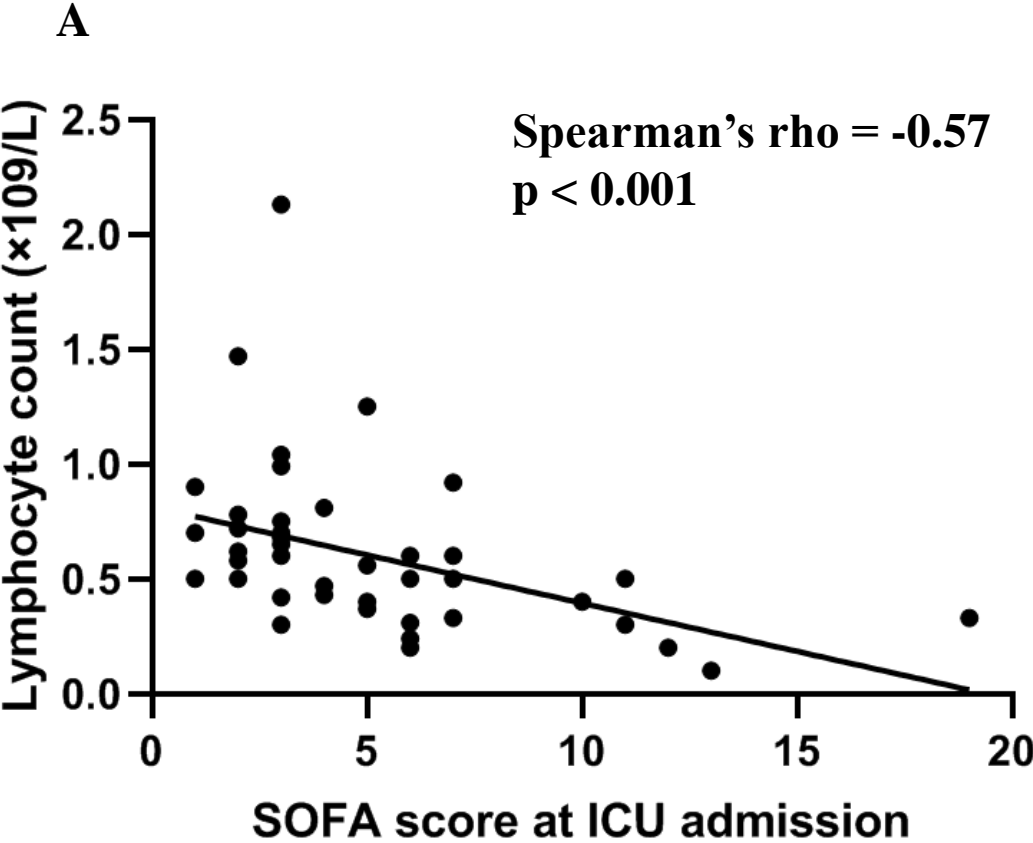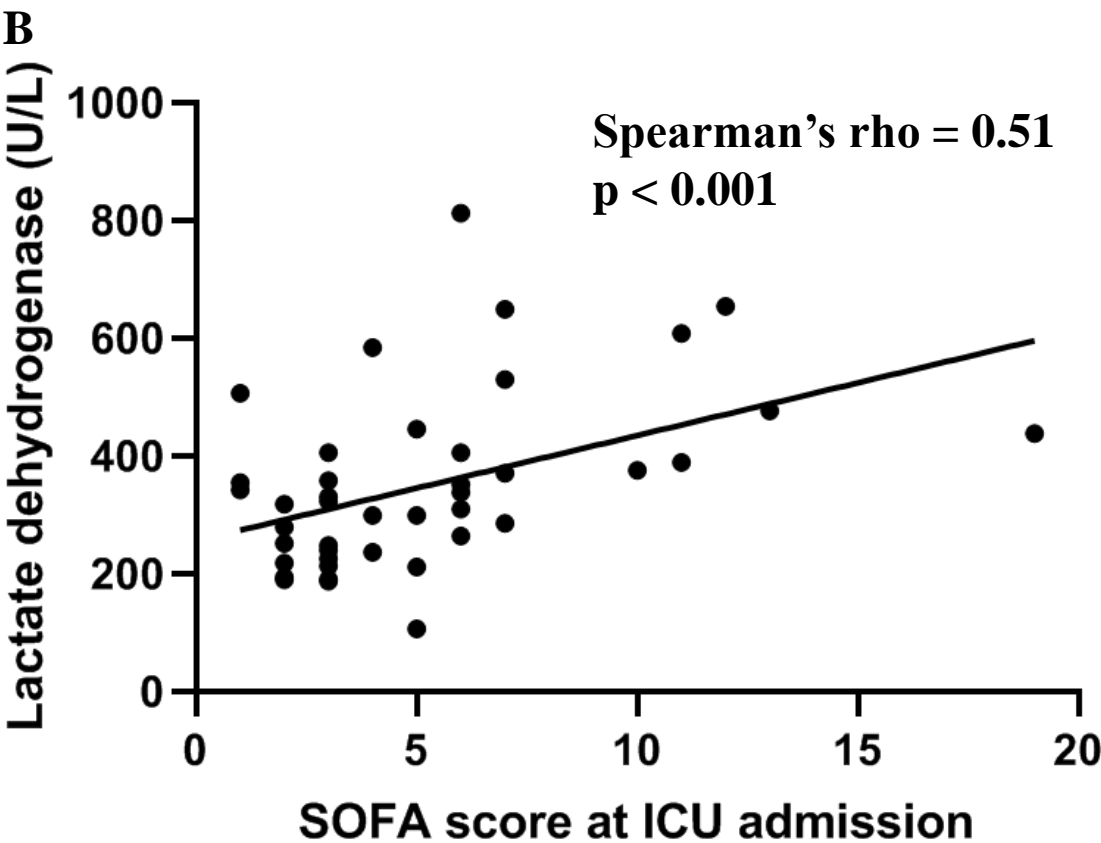

Supplement: Supplementary file 3 [file Image_1.pdf]
